# Stenotrophomonas maltophilia in people with Cystic Fibrosis: a systematic review of prevalence, risk factors and management

**DOI:** 10.1007/s10096-023-04648-z

**Published:** 2023-09-20

**Authors:** Vito Terlizzi, Marta Tomaselli, Giulia Giacomini, Irene Dalpiaz, Elena Chiappini

**Affiliations:** 1 Department of Paediatric Medicine, Meyer Children’s Hospital, IRCCS, Cystic Fibrosis Regional Reference Center, Meyer Children’s Hospital, Florence, Italy; 2 Department of Health Sciences, Anna Meyer Children’s University Hospital, IRCCS, University of Florence, Florence, Italy; 3 Department of Paediatric Infectious Disease, Anna Meyer Children’s Hospital, University of Florence, 50139 Florence, Italy

**Keywords:** Systematic review, Risk factors, Prevalence, Lung function, Antimicrobial therapy

## Abstract

To summarize the current knowledge of the clinical impact of *Stenotrophomonas maltophilia* (*SM*) in cystic fibrosis (CF) patients. A systematic review according to the Preferred Reporting Items for Systematic Reviews and Meta-analyses (PRISMA) guideline recommendations, was performed through searches in PubMed and EMBASE databases, and CF National and International Registries websites from 2000 to 2022. Overall, 184 articles were initially retrieved, out of which 15 were selected and included in the review. Data form 6 Registries and 9 pertinent articles from the references of the studies selected were also considered, resulting in 30 studies in total. The prevalence of *SM* in patients with CF is increasing in Europe while it is declining in North America. The role of chronic colonization of *SM* on lung function and clinical status in CF patients is still under debate. The most recent studies suggested a pathogenic role of *SM* chronic infections in CF patients with an acceleration in lung function decline, an increase in hospitalization rates and an association with co-infection. Reflecting the uncertainty about the role of *SM* in CF, little is available about antibiotic therapeutic strategies for both acute exacerbations and chronic infections. Antimicrobial therapy should be performed in the acute exacerbations, while it may be reasonable to attempt eradication when the first colonization is identified. Nevertheless, it is not established which antibiotic regimen should be preferred, and overtreatment could contribute to the selection of antimicrobial-resistant strains. Further studies are warranted in this regard.

## Introduction

Cystic fibrosis (CF) is the most common autosomal recessive inheritable disease in the Caucasian populations [1]. It is caused by variants in the CF transmembrane conductance regulator (*CFTR*) gene on chromosome 7, encoding for a transmembrane channel involved in the transport of chloride ions [2]. The incidence of CF has traditionally been estimated at 1/2500 live births in the Caucasian population, indeed data from newborn screening reveals a lower incidence nowadays, between 1/3000 and 1/6000 [3]. Clinically, the failure of the transmembrane channel, results in a multisystemic illness with the major involvement of the respiratory system, where CF causes a chronic and progressive obstructive disease. The lung disease in CF represents the main cause of death, giving the multiple bacterial colonization leading to recurrent infections and respiratory insufficiency [4]. People with CF are well known to develop chronic respiratory infections with opportunistic bacterial species, mainly gram negative, including *Pseudomonas aeruginosa* (*PA*)*, Methicillin-resistant or Methicillin-Sensitive Staphylococcus aureus, Burkholderia* species, *Stenotrophomonas maltophilia* (*SM*) *and Achromobacter xylosoxidan*s [5]*.* Although the role of pathogen *PA* is well described in patients with CF, with a prevalence of colonization up to 80% in adults [6], less is known about *SM,* considered an emerging bacterial species in CF.

*SM* is a multidrug-resistant Gram-negative obligate aerobe bacterium commonly found in CF airways which can cause colonization and chronic infection in CF. There is no agreement whether or not *SM* has to be considered a pathogen for lung disease, although most studies described an impact of this species on pulmonary function in patients affected by CF [7].

Defining the role of *SM* as a potential pathogen in CF patients is crucial, as the eventual pathogenicity might require to define safe and effective eradication methods. Distinguishing between chronic infections, acute exacerbations and colonization could be difficult, without a univocal agreement concerning the three definitions. Considering the possible eradication of *SM*, its intrinsic resistance to antibiotics has to be taken into account. The antibiotic drug resistance of *SM* is mainly due to genes encoding multidrug efflux pumps and antibiotic inactivating enzymes [8]. *SM* is intrinsically resistant to many beta-lactam antibiotics and aminoglycosides [9] with common resistance also to levofloxacin and ceftazidime, even if many isolates remain susceptible to trimethoprim-sulfamethoxazole and minocycline^8^. The multidrug resistance makes the identification of a successful eradication strategy challenging, without a consensus on which antibiotic drug should be adopted in case of colonization and pulmonary disease by *SM* in CF [10].

Many aspects of the clinical impact of *SM* in CF are still uncertain: in order to summarize the current knowledge on this issue we conducted a systematic review of the available literature, particularly on: (1) prevalence and risk factors of *SM* in patients with CF, (2) the impact of *SM* infection on lung function and (3) therapeutic options available for acute and chronic infections by *SM*.

## Methods

### Study design

A systematic review of the literature was performed according to the Preferred Reporting Items for Systematic Reviews and Meta-analyses (PRISMA) guideline recommendations [11]. A search of the literature in medical databases, including MEDLINE by PubMed, Cochrane Library and EMBASE, for articles published in English from 2000 to 2022, was performed. Used keywords, limited to Title, were as follows: “(Stenotrophomonas maltophilia) AND (Cystic Fibrosis)”. Duplicates were removed, references of selected articles were included if pertinent and if they fulfilled the inclusion criteria. National and International CF Registries were searched on Google Scholar database.

For completeness, we subsequently expanded the search strings by adding the following keywords: limited to title: "(Stenotrophomonas maltophilia) AND (Cystic Fibrosis) AND (lung function)"; "(Stenotrophomonas maltophilia) AND (Cystic Fibrosis) AND (FEV1)"; "(Stenotrophomonas maltophilia) AND (Cystic Fibrosis) AND (treatment)"; "(Stenotrophomonas maltophilia) AND (Cystic Fibrosis) AND (antibiotic)"; "(Stenotrophomonas maltophilia) AND (Cystic Fibrosis) AND (genotype)"; "(Stenotrophomonas maltophilia) AND (Cystic Fibrosis) AND (phenotype)"; "(Stenotrophomonas maltophilia) AND (Cystic Fibrosis) AND (heterogeneity)". We also extended the search to include: "(Stenotrophomonas maltophilia) AND (lung function)"; "(Stenotrophomonas maltophilia) AND (FEV1)"; "(Stenotrophomonas maltophilia) AND (treatment)"; "(Stenotrophomonas maltophilia) AND (antibiotic)"; "(Stenotrophomonas maltophilia) AND (genotype)"; "(Stenotrophomonas maltophilia) AND (phenotype)"; "(Stenotrophomonas maltophilia) AND (heterogeneity)". After this further search and after removing duplicates, no additional information was found beyond what had already been selected in the previous articles.

### Inclusion and exclusion criteria

The search was restricted to the English language. Articles reporting *SM* prevalence in patients with CF, risk factors for *SM* infection, effect of *SM* on lung function and therapeutic strategies available for *SM* eradication in acute and chronic settings were initially included. Review articles, commentaries, editorials, and letters to the author with no original data were excluded.

### Data extraction

Duplicate publications were removed, then three authors separately (MT, GG and ID) checked the titles and abstracts and removed irrelevant studies according to the inclusion and exclusion criteria. For each article with original data: author, country, year of publication, type of study, type and number of included participants were analysed and summarized in Table 1.

**Table 1:** Original studies included in the review

Author	Year	Country	Type of study	Participants	Sample size
Stanojevic et al. [12]	2013	Canada	Retrospective cohort study	CF^*^ patients (mean age: 11.97 years)	601
Graff et al. [13]	2001	USA	Two identical, randomized, placebo-controlled trials	CF^*^ patient with chronic PA^***^ endobronchial infection. (mean age: 20.8 years)	520 tobramycin inhalation therapy group, 258 patients; placebo group, 262 patients
Talmaciu et al. [14]	2000	USA	Case-control study	CF^*^ patient (mean age: 8.6 years)	58
Denton et al. [15]	1996	UK	Retrospective case-control study	CF^*^ patients (mean age: 11.2 years)	12 cases; 24 controls
Marchac et al. [16]	2003	UK	Case-control study	CF^*^ patients (median age: 22.6 years)	63 cases; 52 controls
Paugam et al. [17]	2010	France	Retrospective cohort study	CF^*^ patients 17 – 65 years (median age: 26 years)	201
Dalbøge et al. [18]	2011	Denmark	Retrospective case-control study	CF^*^ patients (median age: 12.5 years)	278
Waters et al. [19]	2013	Canada	Longitudinal cohort study	CF^*^ patients (median age: 14.7 years)	687
Com et al. [20]	2014	USA	Retrospective cohort study	CF^*^ patients (median age: 12 months)	122
Cogen et al. [21]	2015	USA	Retrospective observational study	CF^*^ patients < 12 years of age, *PA*-negative (mean age: 5.7 years)	946
Barsky et al. [22]	2017	USA	Longitudinal retrospective study	CF^*^ patients (mean age: 17.4 years)	88
Poore et al. [23]	2022	USA	Retrospective cohort study	CF^*^ patients (mean age: 12.4 years)	294
Berdah et al. [24]	2018	France	Case-control retrospective study	CF^*^ patients (mean age: 10.1 years)	23 cases; 23 controls
Vidigal et al. [25]	2014	Germany	Comparative genomic and phenotypic analysis	90 SM^**^ strains from 19 CF^*^ patients	
Pompilio et al. [26]	2016	Italy	Comparative genomic and phenotypic analysis	13 SM^**^ strains from a CF^*^ patient	
Esposito et al. [27]	2017	Italy	Comparative genomic and phenotypic analysis	91 SM^**^ strains from 10 CF^*^	
Alcaraz et al. [28]	2021	Brazil	Comparative genomic and phenotypic analysis	11 SM^**^ strains from a CF^*^ patient	
San Gabriel et al. [29]	2004	USA	Survey of SM^**^ isolates	955 SM^**^ strains from 673 CF^*^ patients	
King et al. [30]	2010	USA	Antimicrobial activity tested	Isolates from the sputum of CF^*^ patients	486
Goss et al. [31]	2021	USA	Multicenter, randomized, controlled clinical trial	CF^*^ patients (median age: 29.3 years)	982
Capaldo et al. [32]	2020	France	Retrospective cohort study	CF^*^ patients (mean age: 24.4 years)	90
Psoter et al. [33]	2017	USA	Retrospective study	CF^*^ patients (mean age: 24.5 months)	4552
Goss et al. [34]	2004	USA	Retrospective cohort study	CF^*^ patients aged >6 years. (median age: 13.8 years)	20755
Waters et al. [35]	2012	Canada	Retrospective cohort study	CF^*^ patients (median age: 19.4 years)	440

*CF: cystic fibrosis

**SM: Stenotrophomonas maltophilia

***PA: Pseudomonas aeruginosa

### Quality assessment

For observational studies, adherence to Strengthening the Reporting of Observational Studies in Epidemiology (STROBE) recommendations [36] was assessed and reported in Table 2. The results of the assessment suggest that the overall quality of studies included in the systematic review was medium. Particularly, most of them did not evaluate bias of the study and did not give an adequate characterization of the quantitative variables and of the data sources. However, almost all studies clearly defined study design, setting, outcome data and adequately discuss the key results.

**Table 2:**
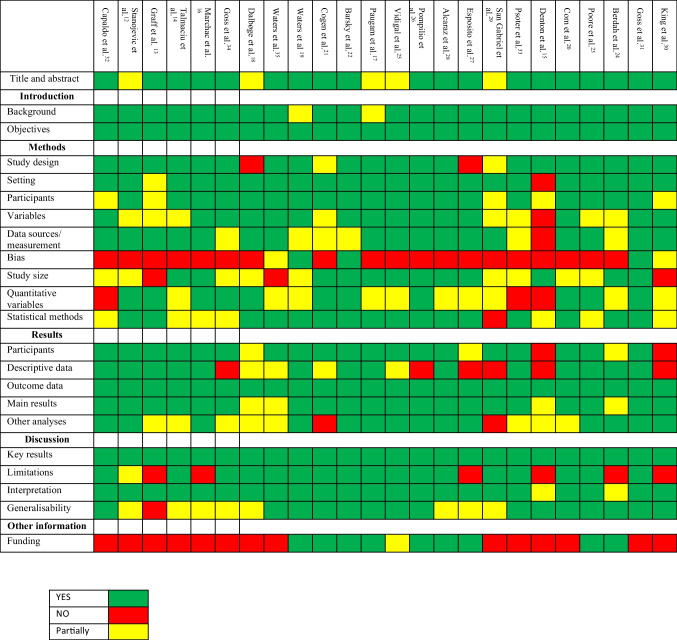
Adherence to STROBE recommendations

## Results

### Study characteristics and quality

Overall, 184 records were initially identified and duplicates were removed. One hundred and five articles were screened, of which 70 were excluded by title and abstract because they didn’t match the inclusion criteria. The remaining 35 records were analysed in full text and we selected 15 articles with original data focused on clinically relevant aspects such as S*M* prevalence in patients with CF, risk factors for *SM* infection, effect of *SM* on lung function and therapeutic strategies.

**Fig. 1:**
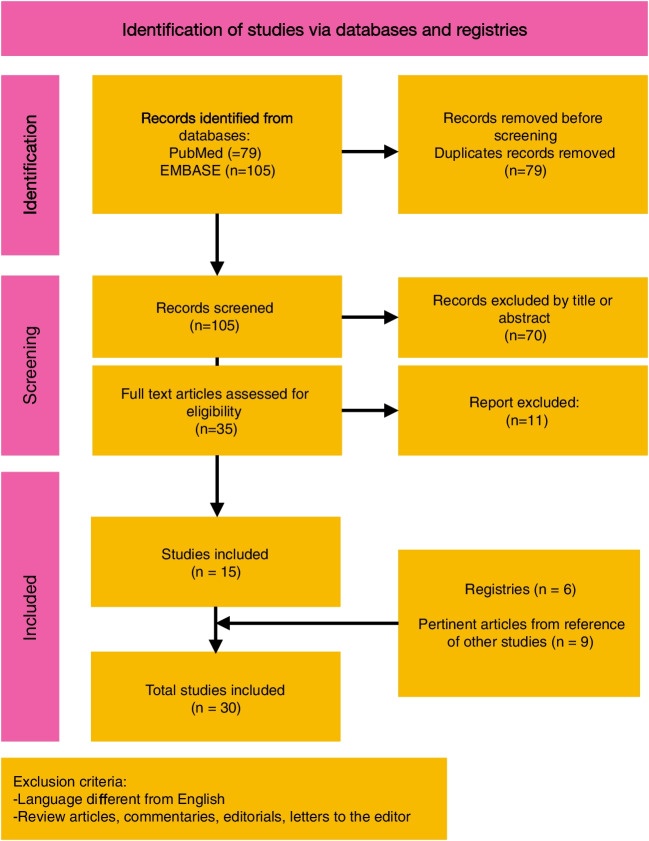
Flow diagram of literature search and data extraction

Out of the resulted articles, the 2021 Cystic Fibrosis Foundation Patient Registry (CFFPR) [37], the 2021 European Cystic Fibrosis Society Patient Registry (ECFSR)[38], the 2020 Italian Cystic Fibrosis Registry (ICFR) [39], the 2021 French Cystic Fibrosis Registry (FCFR) [40], the 2021 Australian Cystic Fibrosis Data Registry (ACFDR)[41], the 2021 Canadian Cystic Fibrosis Registry (CCFR) [42], were included in the Review. Nine pertinent articles from the references of the studies selected were also considered. Figure 1 shows the flow diagram of literature search and data extraction.

Characteristics of the original articles included in the review are summarized in Table 1.

### Study outcomes

The original articles and the registries included in the systematic review were related to 4 mail topics: (1) prevalence and risk factors for *SM* infection and colonization, (2) impact of *SM* on lung function in patients with *SM*, (3) genotype and phenotype heterogeneity of *SM*, (4) antimicrobial therapy available against *SM* infection.

Main outcomes of each original article included in the review are summarized in Table 3 and discussed more in detail in the dedicated paragraphs in the Discussion section.

**Table 3:** Main outcomes of the original studies included in the systematic review

Author	Main outcome				
	1. Prevalence				
Capaldo et al. [32]	Increasing trend in the prevalence of SM* in cystic fibrosis patients				
Psoter et al. [33]	No seasonal variation for SM* infection				
	2. Suggested risk factors				
Stanojevic et al. [12]	Lung function decline; younger age				
Graff et al. [13]	Oral antibiotics				
Talmaciu et al. [14]	Exposure to antibiotics; compromised clinical status				
Denton et al. [15]	Exposure to antibiotics; previous hospitalization				
Marchac et al. [16]	Exposure to antibiotics; exposure to oral steroids; *Aspergillus fumigatus* co-infection				
Paugam et al. [17]	*Aspergillus fumigatus* co-infection				
	3. SM* and Lung function				
	Impact on lung function	No impact on lung function	Decrease in FEV1	Increase in hospitalization, mortality, lung transplantation	Fungal co-infection
Goss et al. [34]		x			
Dalbøge et al. [18]	x		x		
Waters et al. [35]		x			
Waters et al. [19]				x	
Com et al. [20]	x		x	x	
Cogen et al. [21]	x		x		
Barsky et al. [22]	x		x		
Poore et al. [23]	x				x
Berdah et al. [24]	x		x		
	4. Genotype and phenotype heterogeneity of SM*				
Vidigal et al. [25]	• High genotype and phenotype heterogeneity of SM* as expression of adaptability of the bacteria • No evidences about the impact of the heterogeneity on lung function				
Pompilio et al. [26]					
Esposito et al. [27]					
Alcaraz et al. [28]					
	5. Antimicrobial therapy against SM*				
Waters et al. [35]	Impact on lung function:		No impact of antibiotic therapy targeting SM during pulmonary exacerbations in patients with chronic SM infection did not affect the degree of FEV1 recovery or the time to subsequent exacerbation.		
Esposito et al. [27]	Suggested antimicrobial drug:		Minocycline, doxycycline, trimethoprim-sulfamethoxazole		
San Gabriel et al. [29]	Suggested antimicrobial drug:		Trimethoprim-sulfamethoxazole, ticarcillin-clavulanate, doxycycline		
King et al. [30]	Suggested antimicrobial drug:		Aerosolized levofloxacin in chronic SM infections		
Goss et al. [31]	Duration of antimicrobial therapy:		Same outcome** in acute exacerbation in CF patient for 10, 14 and 21-day regimens		

*SM: *Stenotrophomonas maltophilia*

**Outcome: FEV1 improvement

**Fig. 2:**
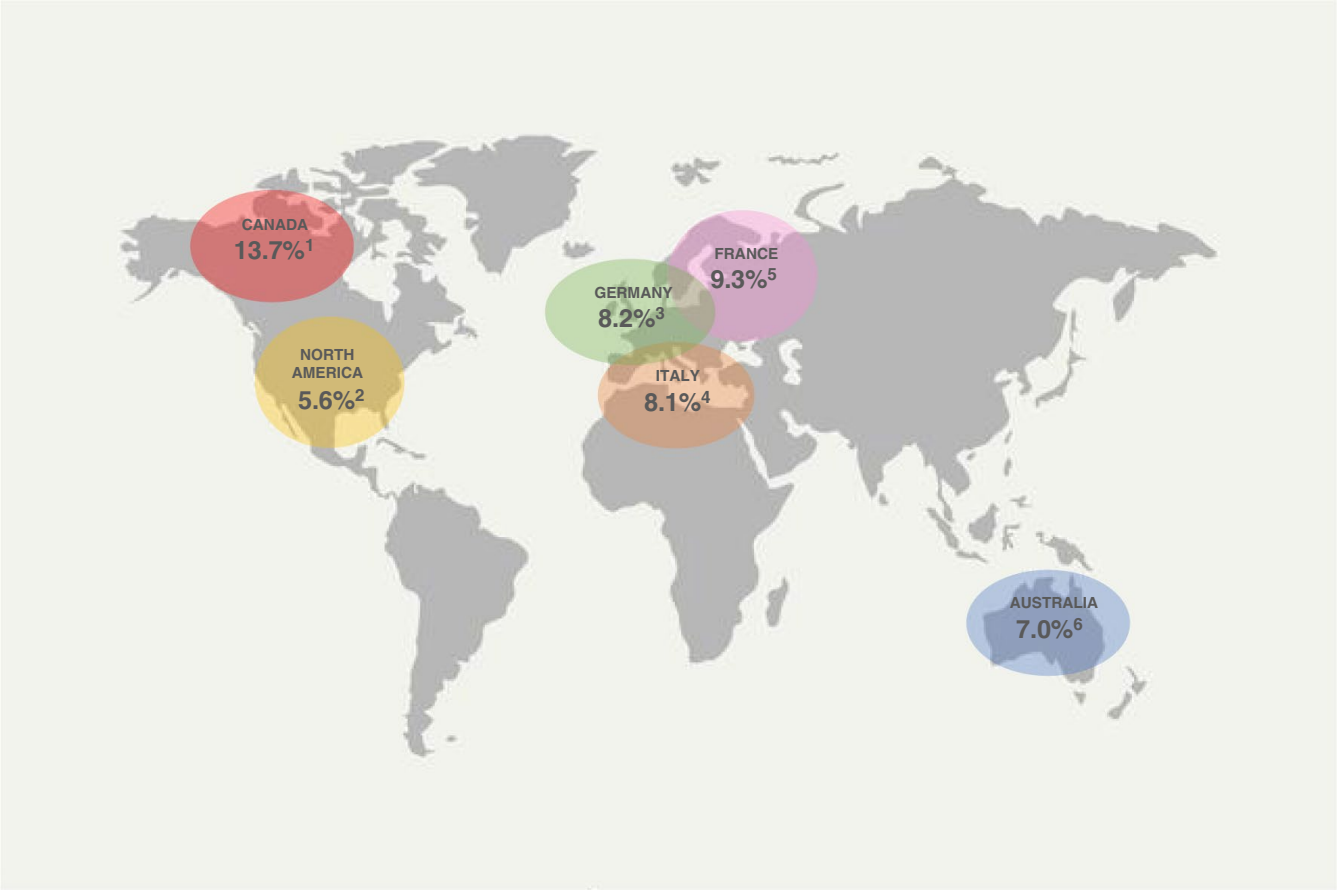
Prevalence of Stenotrophomonas maltophilia in adults and children with Cystic Fibrosis by Country. (1) Data from the 2021 Canadian Cystic Fibrosis Registry; (2) data from the 2021 Cystic Fibrosis Foundation Patient Registry; (3) data from the 2021 European Cystic Fibrosis Society Patient Registry; (4) data from the 2020 Italian Cystic Fibrosis Registry; (5) data from the 2021 Australian Cystic Fibrosis Data Registry

Prevalence of *SM* in patients with CF was analysed based on the national and international registries from countries with high prevalence of CF. Some registries, such as the French and the North America’s ones [37, 40], showed a declining trend in prevalence of SM, on contrast the ICFR reported an increasing in *SM* prevalence in patients with CF [39]. Differences in prevalence of *SM* infection by country are represented in Fig. 2. Which factors could play a role as risk factor for *SM* infection is unclear, although some studies suggested: younger age, lung function decline, use of antimicrobial drugs, exposure do oral steroids and *Aspergillus fumigatus* co-infection [12–17].

The impact of *SM* infection on lung function in patients with CF is still under debate. Most studies included in the systematic review related to this topic (seven out of nine articles) showed a worst pulmonary outcome in CF patients with *SM* infection, particularly with a higher decrease in Forced Expiratory Volume in the 1st second (FEV1), increase in hospitalization, in mortality and an in need for lung transplantation [18–24]. A higher proportion of fungal co-infections, mainly by *Aspergillus fumigatus*, was also reported in CF patients with *SM* infection [23].

All the articles about genotype and phenotype heterogeneity of *SM* reported a high heterogeneity, although the role of this heterogeneity in the pulmonary outcome has not been investigated [25–28].

No antimicrobial drug nor antimicrobial regimen’s duration for treatment of *SM* infections, both acute and chronic, has been established. Related to the antimicrobial drugs, minocycline, doxycycline, trimethoprim-sulfamethoxazole, ticarcillin-clavulanate and aerosolized levofloxacin were suggested by the original articles included in the systematic review [27, 29, 30]. The antibiotic strategies available against *SM* infection in CF are summarized in Table 4. Very little is available about the antimicrobial regimen’s duration, although no differences from a 10-day regimen to a 21-day regimen was reported [31].

**Table 4:** Available antimicrobial drugs with activity against *Stenotrophomonas maltophilia*

Antibiotic	Dosage
Trimethoprim-sulfamethoxazole	8-12 mg/kg/die IV^*^ divided every 8 hours or every 12 hours
Levofloxacin	750 mg IV^*^/po^**^ every 24 hours
Minocycline	200 mg IV^*^/po^**^ every 12 hours
Tigecycline	200 mg IV^*^ (first dose) then 100 mg IV^*^ every 12 hours
Cefiderocol	2 g IV^*^ every 8 hours infused over 3 hours
Ceftazidime-avibactam	2.5 g IV^*^ over 3 hours every 8 hours
Aztreonam	2 g IV^*^ over 2 hours every 8 hours

^*^Intravenous dosing

^**^Oral dosing

## Discussion

### Prevalence and risk factors

The reported prevalence of *SM* among patients with CF is various, with many differences mainly depending on the country. For the 2021 CFFPR Annual Data Report, including CF patients from North America, the prevalence of the bacteria was 5.6%, declining from the reported prevalence of 12.7% and 13.1% respectively in 2006 and 2016 [37]. Also the 2021 CCFR reported a decrease in *SM* prevalence in the last few years, from 14.2% in 2017 to 13.7% in 2021 [42, 43]. Interestingly, in contrast with these results, in France an increasing trend in the prevalence of colonization by *SM* in CF patients has been reported, from 4.7% in 1999 to 10.5% in 2016 [32]. The proportion of colonization by *SM* in CF patients in France is still decreasing, as the 2021 FCFR reported 9.3% of patients to be colonized [40]. According to the 2021 ECFSR data, the prevalence of the *SM* infections was 6.6% in children and 7.7% in adults, considering both chronic and not chronic or intermittent infections. The highest proportions were observed in Northern Europe, reaching 25.0% in the paediatric population in Iceland [38]. Differently from the proportion reported by the 2021 ECFPR, the 2021 ACFDR reported a higher prevalence of *SM* in children than in adults affected by CF, respectively 7.6% and 6.5% [41]. In Italy in 2020 the prevalence of *SM* reported by the ICFR was 7.6% in the adult population and 8.6% in children. The data reported by the ICFR, in contrast with that of North America, showed an increasing prevalence of *SM*, with and increasing from 2.9% in 2018 to 7.6% in 2020 in adults and from 2.6% in 2018 to 8.6% in 2020 in paediatrics [39].

Figure 2 summarizes the average *SM* prevalence in adults and children affected by CF, considering the proportions reported by the Registries included in the review.

While a seasonal variation for some common bacterial pathogens in CF patients has been described, as for *PA,* methicillin-susceptible *Staphylococcus aureus*, *Achromobacter xylosoxidan*s and *Haemophilus influenzae*, such variation was excluded for *SM* [33].

The differences in prevalence are likely due to variations in local ecology [32], even if some other factors influencing the acquisition of the infection might play a role. Little is known about factors which could impact on the colonization of *SM*, and consequently may influence the prevalence in patients with CF. Firstly, some studies described an association between the severity of lung disease and the acquisition of *SM* infection, with a higher risk in patients with a faster decline in FEV1 [12]. It is debated whether the use of antibiotics could represent a risk factor for the infection. Some studies reported a higher risk of *SM* isolation in case of antibiotic therapy [13, 14], nevertheless, other authors described exposure to antibiotic courses as a protective factor through the preservation of lung function [12]. Particularly, Denton et al. described an increase in *SM* isolation in patients who received anti-*PA* antibiotic courses, suggesting that the treatment of common infections in CF patients could raise the risk of colonization by *SM* [15]*.* A study by Marchac et al. described an association between the isolation of *Aspergillus fumigatus* and subsequent *SM* infection, although this finding has not been well supported [16]. In agreement with Marchac’s study, Paugam et al. observed a higher proportion of *SM* colonization in CF patients with *Aspergillus fumigatus* [17]*.*

### Effect of chronic Stenotrophomonas maltophilia infection on lung function

The effect of chronic colonization of *SM* on lung function and clinical status in CF patients is still unclear. Goss et al. analysed data from the CF Foundation National Patient Registry (CFNPR) registers from 1994 to 1999. In this extensive cohort study on 2739 CF patients, there was no association between *SM* and a decrease of lung function after controlling for confounders (age, sex, weight, height, pancreatic insufficiency, *PA* and *Burkolderia cepacia* colonization, use of intravenous antibiotics) [34]. A subsequent cohort study from 2008 to 2009 compared 82 CF patients with at least one positive culture of *SM* to a CF control group with no chronic gram-negative infections. In this study, patients with *SM* positive cultures every month for 6 consecutive months or, less often, when combined with an increase in number of specific, precipitating antibodies were defined as chronically infected. They found that patients who had been chronically infected with *SM* for at least 2 years, had a significantly larger decline in lung function, demonstrated as change in FEV1% of predicted value per year. However, no change was detected in the rate of FEV1 decline when those patients were compared to themselves in the previous 3 years before they became chronically infected [18]. A similar retrospective cohort study showed that chronic *SM* status (defined as 2 or more positive sputum or bronchoalveolar cultures in the previous 12 months) does not affect FEV1 recovery and *SM* antibiotic treatment does not influence the recovery or the gain in FEV1 after a pulmonary exacerbation [35]. In contrast, the same group found increased rates of mortality and lung transplantation among patients with *SM* chronic infection, although this effect was no longer significant in a time-varying model that includes lung function [19].

Recently, some observational studies suggested that *SM* chronic infection may be associated with worse respiratory outcomes and accelerated lung function decline. In a retrospective review of medical records with CF in the USA, Com et al. compared children with low and high initial FEV1, in order to analyse their baseline characteristics. The authors described a significant correlation between low initial FEV1 measurements and positive respiratory culture for *SM* (*p*<0.05) [20]. In 2015, Cogen et al. in a multicenter longitudinal observational study, in order to identify a high-risk group in *PA*–negative and ≤12 years of age children with CF, described *SM* as a risk factor for FEV1 decline [21]. A subsequent longitudinal retrospective study of 88 patients demonstrated that the acquisition of *SM* is associated with an acceleration in lung function decline. More interestingly, the effect persisted after controlling for confounders. In this study, chronic infection was defined as two or more positive cultures within a 12-month time period following acquisition, otherwise infection was classified as intermittent. Interestingly, both the intermittent and chronic subgroups were associated with lung function decline, and the change in rate of decline did not significantly differ between them. Chronic *SM* infection was also associated with an almost twofold increase in mean annual hospitalizations (*p*=0.007) [22].

In a recent retrospective study Poore et al. noticed an association between *SM* colonization and frequent fungal infection, especially *Aspergillus* (70% of fungal positive cultures in this cohort). Furthermore, they found that patients with *SM* and frequent fungal isolation had lower average lung function by almost 10% compared to controls [23].

We found several limitations in these studies, such as small cohorts of patients, type of study design (lack of prospective studies) and different clinical characteristics among patients included. Moreover, the definition of chronic colonization is based on different criteria among the studies, which makes them barely comparable. However, the most recent studies suggest a more active role of SM in influencing the progression of lung disease rather than simply being an indicator of disease severity. This can probably be explained by the fact that *SM* was considered a classical but infrequent bacterium in CF patients until the 2000's, but its incidence appears to be increasing in recent decades [24].

### Genotypic and phenotypic heterogeneity of Stenotrophomonas maltophilia

While the genetic adaptations and resulting phenotypic variations in *PA* and *Staphylococcus aureus* colonization of CF lungs are well-documented, the specific adaptive characteristics of *SM* that contribute to its persistence in CF patient only recently gained interest among many authors[25–28]. Genetic studies have revealed significant genotypic diversity within *SM* chronically infected CF patients. Multiple strains of *SM* can coexist within an individual patient, suggesting ongoing acquisition and colonization events [26, 28]. Genotyping techniques, such as pulsed-field gel electrophoresis and multilocus sequence typing have provided insights into the clonal relatedness and genetic variation among different isolates. The phenotypic variability is observed in various aspects, including antibiotic resistance patterns, biofilm formation, and virulence factors.

In a study by Vidigal et al. genotypic diversity, mutation frequency, and antibiotic resistance were examined in 90 *SM* isolates from 19 CF patients with chronic colonization [25]. The findings revealed that *SM* undergoes significant genetic diversity during chronic CF lung infection, although a decreased mutation rate was observed in the later isolates. In a more focused investigation, Pompilio et al. 2016 evaluated 13 *SM* strains isolated from a single CF patient with chronic infection over a 10-year period [26]. They examined various traits including growth rate, biofilm formation, motility, mutation frequencies, antibiotic resistance, and pathogenicity. The results demonstrated that *SM* adaptation led to increased antibiotic resistance but decreased in vivo pathogenicity and biofilm formation. However, it is important to note this study's limitation of only considering one chronically infected patient. Interestingly, according to Esposito et al. and Alcaraz et al. the wide range of phenotypes exhibited by *SM* strains, only marginally correlates with the distribution of mutations across their genomes [27, 28].

These studies collectively emphasize the remarkable adaptability of *SM* during chronic infection in CF patients. This heterogeneity likely arises from the microorganism's need to adapt to a highly challenging CF lung environment, while facing diverse selection pressures based on the host's unique conditions. The mechanisms that drive the development of high genomic heterogeneity, resulting in a wide range of phenotypes, is still unclear and further studies are needed in order to better understand it [27]. Although the discussion of this topic is beyond the scope of our review, which is focused on clinical aspects of *SM* in CF, it will be important to clarify the mechanisms of development of genotypic and phenotypic heterogeneity, giving the possible impact on diagnosis, treatment, and infection control strategies.

### Treatment of *Strenotrophomonas maltophilia* acute and chronic infections

At present there are no clear guidelines regarding the management of *SM* in people with CF, as literature is poor and it is still uncertain if both the treatment of acute exacerbation and the long-term suppressive therapy are effective.

A Cochrane Intervention Review by Amin et al. was conducted to assess the effectiveness of antibiotic treatment in people with CF, primarily in the setting of acute pulmonary exacerbations and then in chronic colonization of *SM*. However, there was no evidence since no randomized control trial met the inclusion criteria for the review [10].

The objective of administering antibiotics during a CF pulmonary exacerbation is twofold: to decrease the bacterial presence in the airways, potentially eliminating the bacteria altogether, and to reduce inflammation, consequently enhancing lung function and extending the period before another exacerbation occurs [44].

A retrospective cohort study showed that antibiotic therapy targeting *SM* during pulmonary exacerbations in patients with chronic *SM* infection did not affect the degree of FEV1 recovery or the time to subsequent exacerbation [35]. It is worth noting, however, that the majority of patients received treatment with a single antimicrobial drug targeting SM, resulting in successful elimination of *SM* from the airways in only a quarter of chronic SM pulmonary exacerbations. It is widely known that *SM* exhibits intrinsic resistance to a wide range of antimicrobial agents, and it is often recommended to employ a combination of antibiotics to effectively treat *SM* infections. Even the authors suggested that the antimicrobial monotherapy may not be sufficient.

*SM* is a multidrug-resistant opportunistic bacteria and can rapidly develop antimicrobial resistance mutations [45]. Trimethoprim-sulfamethoxazole has been historically considered the first line of treatment for *SM* infections due to high susceptibility rates and large clinical experience [45–47]. According to Esposito et al. the most effective antibiotics against *SM* were minocycline, doxycycline and trimethoprim-sulfamethoxazole, showing comparable susceptibility rates [27]. Whereas San Gabriel et al. demonstrated that in vitro, *SM* appears to be most susceptible to trimethoprim-sulfamethoxazole, ticarcillin-clavulanate and doxycycline [29].

Thus, treatment of *SM* infections in CF patients poses a big challenge and until further evidence of the role of antimicrobial regimes is available, clinicians need to decide on clinical judgement on a case-by-case basis.

The Infectious Diseases Society of America (IDSA) provided guidance for *SM* infections management in CF and non-CF patients, consisting of "suggested approaches" based on clinical experience, expert opinion, and a review of the available literature [48]. In case of moderate to severe infections, and also considering the multiple mechanism of antibiotic resistance, they recommended a combination therapy. They suggested 3 approaches: (1) the use of combination therapy, with trimethoprim-sulfamethoxazole and minocycline as the preferred combination; (2) the initiation of trimethoprim-sulfamethoxazole monotherapy with the addition of a second agent (minocycline [preferred], tigecycline, levofloxacin, or cefiderocol) if there is a delay in clinical improvement with trimethoprim-sulfamethoxazole alone; (3) the combination of ceftazidime avibactam and aztreonam, when intolerance or inactivity of other agents are anticipated. For mild infections and polymicrobial infections where the role of SM is unclear, they suggested monotherapy with trimethoprim-sulfamethoxazole, levofloxacin, minocycline, tigecycline or cefiderocol. Table 4 reports the suggested dosages for antimicrobial therapy.

The multicenter randomized controlled clinical trial STOP2 evaluated the antimicrobial therapy duration during acute exacerbations in CF adults, regardless of the bacterial species involved. The outcome was similar for the 10 day, the 14 day and the 21 day regimens [31].

No suppressive therapy for CF patient with *SM* chronic infection is available, despite aerosolized levofloxacin as a potential future strategy [30].

## Study limitations

Our review has some limitations. Methodological issues were frequent among the included studies, such as small cohorts of patients, lack of prospective studies and different clinical characteristics among patients included. Especially, regarding therapeutic strategies, literature is very poor and there is no agreement among authors. This fact, along with the overall scarcity and heterogeneity of data, precluded us from making a metanalysis of the selected studies.

## Conclusion

Over the past two decades, *SM* has emerged as a significant pathogen in CF patients, and its incidence appears to be on the rise. Regardless of the specific characteristics of the infection, *SM* is now recognized as a detrimental pathogen that can have a substantial impact on lung function in individuals with CF. Consequently, there is a pressing need to establish suitable strategies for eradicating *SM*, mirroring the recommended approach for initial *PA* infections. To address this, the standardization of the definition for chronic and intermittent *SM* infection will be crucial. Developing a consensus on the criteria and parameters that differentiate the two types of infection could make accurate diagnosis, treatment and monitoring of *SM* infections easier. Standardization will also promote consistency in research findings and allow for better comparison of results across studies.

Recent studies described the pathogenic role of *SM* infections in CF with an acceleration in lung function decline, an increase in hospitalization rates and an association with co-infection, such as fungal infections. Therefore, we suggest an antimicrobial therapy for acute exacerbations. However, it may be reasonable to attempt eradication even when first colonization is identified.

Despite most authors suggesting trimethoprim-sulfamethoxazole as first-line treatment, considering the multidrug resistance exhibited by *SM*, combination therapy involving two other agents may be recommended. In conclusion, future randomized clinical trials are needed in the adult and paediatric populations to select the proper treatment, both for *SM* acute and chronic infections.

## Abbreviations

PA: Pseudomonas aeruginosa
SM: Stenotrophomonas maltophilia
CF: Cystic Fibrosis
CFFPR: Cystic Fibrosis Foundation Patient Registry
ECFS: European Cystic Fibrosis Society
ICFR: Italian Cystic Fibrosis Registry
FCFR: French Cystic Fibrosis Registry
ACFDR: Australian Cystic Fibrosis Data Registry
CCFR: Canadian Cystic Fibrosis Registry
FEV1: Forced Expiratory Volume in the 1st second
